# Hospital-Based Emergency and Trauma Care—The Expanding Epicenter of the US Healthcare Delivery System

**DOI:** 10.3390/healthcare13121424

**Published:** 2025-06-13

**Authors:** Glenn Melnick

**Affiliations:** Price School of Public Policy, University of Southern California, Los Angeles, CA 90089-0626, USA; gmelnick@usc.edu

**Keywords:** trauma centers, emergency departments, emergency and trauma utilization, hospital emergency department capacity, hospital trauma center capacity

## Abstract

**Background/Objectives:** This study investigates the evolution of hospital capacity and utilization in California between 2003 and 2023, focusing on emergency departments (EDs) and trauma centers (TCs). We seek to document structural changes in the healthcare delivery system with respect to hospital-based emergency and trauma services. **Methods:** This analysis examines changes in population demographics, hospital resources, and patient utilization patterns across facility types. Given the significant increase in the proportion of the population aged 65+ and the documented higher use of emergency and trauma services by this population, we expected to observe an expansion in ED and trauma service capacity and utilization. **Results:** Utilizing a comprehensive dataset of California general acute care hospitals over this 20+ year period, our descriptive analysis reveals major shifts in the healthcare delivery system, notably the increased prominence of hospitals with EDs, particularly those designated as trauma centers. Findings indicate that, while the overall number of hospitals and licensed beds has slightly decreased, facilities with EDs, especially trauma centers, have increased their capacity and manage a greater proportion of inpatient admissions and ED visits. **Conclusions:** The increase in ED visits and inpatient admissions at trauma centers, contrasted with decreases in both capacity and utilization at non-trauma hospitals, indicates a significant restructuring of the health delivery system with significant implications for healthcare policy, financing, operations, and affordability. The high and increasing percentage of inpatient admissions originating from hospital EDs and from hospitals with trauma centers suggests a need for policies that foster integration between ED and inpatient care and the broader healthcare delivery system, while at the same time managing the increase in prices and costs associated with growing emergency services utilization. Further research is needed to explore the implications of these trends, particularly concerning their impact on the affordability of healthcare in the US.

## 1. Introduction

The hospital sector is central to the U.S. healthcare system, serving as a primary access point for diverse medical needs and representing a significant portion of national healthcare expenditure, reaching trillions of dollars in 2023 [1]. Emergency departments (EDs) and hospital-based trauma centers (TCs) are critical to this sector. EDs are often the initial contact for acute illnesses or injuries, acting as a vital safety net, especially for vulnerable populations [2,3]. Trauma centers are specialized facilities for managing severe life-threatening injuries, crucial for improving patient outcomes. Understanding trends in ED and trauma center capacity and utilization is essential, as these trends reflect evolving healthcare needs, including changes in disease and injury patterns and access to hospital-based emergency care, as well as, increasingly, the affordability of healthcare in the USA. Monitoring hospital-based emergency and trauma center capacity, along with utilization, helps to monitor health system performance and informs resource allocation and policy planning, especially with respect to evolving alternative payment models such as Value-Based Care (VBC) [4]. The findings from this study will provide valuable insights for policymakers, hospital administrators, and healthcare professionals seeking to optimize healthcare delivery in California, with likely applications to the US healthcare delivery system as a whole.

## 2. Materials and Methods

This paper analyzes comprehensive hospital data to document changes in the capacity and utilization of hospitals in California between 2003 and 2023. This study accesses publicly available data from the California Department of Healthcare Access and Information (HCAI), which mandates reporting from all hospitals in California [5]. Each hospital in California submits detailed financial and utilization data to HCAI on an annual basis. The data are self-reported by each hospital to HCAI. Each hospital’s self-reported data are reviewed by HCAI for completeness and consistency with regulations. HCAI returns reports to a hospital for editing and or revision if the hospital’s report does not pass reviews by HCAI. In terms of data project management, this study accessed hospital-level data for all general acute care (GAC) hospitals for 2003 and 2023 to create a master file for the analyses. The GAC study sample includes hospitals providing a wide range of medical and surgical services with inpatient capacity, and includes children’s hospitals but excludes psychiatric and other specialty hospitals, generating the following sample sizes: n = 398 GAC hospitals in 2003 and n = 379 GAC hospitals in 2023. Individual hospital-level data were then aggregated across all GAC hospitals and, for some analyses, were aggregated in sub-samples based on hospital characteristics. Our analytical approach employs descriptive analyses to compare data from 2003 and 2023. Our descriptive statistical methods are similar to previously published studies in Healthcare [6,7,8,9] that also analyzed aggregate-level data that are similar to our data. We used SAS 9.4 and Excel software for our statistical analyses to generate summary statistics (totals and percentages) across all hospitals and specific sub-samples in each of the study years, 2003 and 2023. Additionally, demographic data for the years 2003 and 2023 are drawn from the State of California Office of Finance.

## 3. Variable Construction

The hospital capacity and utilization variables were each constructed as follows:
**Variable****Construction****Demographics and Hospital ED and Trauma Center Capacity**
Population—total (millions)Sourced from California Department of Finance demographic records for 2003 and 2023.Population, age 65+ total (millions)Sourced from California Department of Finance demographic records for 2003 and 2023.Population, age 65+, percentage total of populationCalculated as (population, age 65+ total/population—total) × 100 for 2003 and 2023.Hospitals—number (all GAC, with EDs, with EDs without TC, with EDs and TC)Counts for 2003 and 2023, classifying hospitals based on reported ED and trauma designation.Licensed beds—number (all GAC, with ED, with ED without TC, with ED and TC)Aggregated licensed beds for hospitals in specified categories for 2003 and 2023.ED stations—number (all with ED, with ED without TC, with ED and TC)Aggregated ED station for hospitals in specified categories for 2003 and 2023.Percentage hospitals with ED or ED + trauma center (various sub-types)Calculated ratios based on counts of hospitals, beds, and ED stations for 2003 and 2023 (e.g., % all beds in hospitals with trauma centers = (licensed beds…/licensed beds…) × 100).% changeCalculated as ((2023 value − 2003 value)/2003 value) × 100 for each variable category.**Hospital Inpatient and ED Utilization**
Total inpatient admissions (all GAC, with EDs, with EDs without TC, etc.)Aggregated inpatient admissions for hospitals in specified categories for 2003 and 2023.ED visits (with EDs, with EDs without TC, with EDs and TC)Aggregated ED visit for hospitals in specified categories for 2003 and 2023.ED visits resulting in IP admission (with EDs, with EDs without TC, etc.)Aggregated counts of ED visits leading to admissions for hospitals in specified categories for 2003 and 2023.% change—2003 vs. 2023Calculated as ((2023 value − 2003 value)/2003 Value) × 100 for each utilization category.**Capacity and Utilization in Hospitals with Trauma Centers**
Level trauma designationCategories classifying hospitals based on official EMSA definitions (Level I, II, III, IV, and various pediatric combinations).Number of hospitalsCounts linked with EMSA trauma designations for 2003 and 2023 for each trauma designation category.ED stationsAggregated ED station for hospitals in each trauma designation category for 2003 and 2023.ED visits total Aggregated total ED visits for hospitals within each trauma designation category for 2003 and 2023.% changes—2003 vs. 2023Calculated as ((2023 value − 2003 value)/2003 Value) × 100 for number of hospitals, ED stations, and ED visits total within each trauma category.**ED/Inpatient Admission Rates by Trauma Level**
Percentage of total ED visits resulting in inpatient admissionCalculated for each trauma category as (ED visits resulting in IP admission/total ed visits) × 100, using HCAI data.Percentage of total inpatient admissions via EDsCalculated for each trauma category as (ED visits resulting in IP admission/total inpatient admissions for that category) × 100.Level of trauma designation Categories (Level I, II, III, IV, pediatric combinations) based on EMSA classifications, used for grouping hospitals to calculate the percentages.**Statewide Admission Shares**
Percentage of total statewide inpatient admissionsCalculated for three hospital groups (with EDs and TCs; with EDs without TCs; without EDs) for 2003/2023 as (total inpatient admissions for group/total statewide inpatient admissions) × 100.

## 4. Trauma Center Level Designation Definitions

An important aspect of this study is its analysis of capacity and utilization within hospitals with designated trauma centers. Hospitals apply for and are designated by regulatory agencies based on their capabilities, staffing, and resources [10]. Trauma center designation levels refer to a classification system used by regulators to categorize hospitals based on their ability to provide care for traumatic injuries and other traumatic emergencies.
**Level****Trauma Center Level Designation Definitions****Level I**Highest level of trauma care; 24 h in-house coverage by general surgeons and prompt availability of care in various specialties; referral resource for communities in nearby regions; provides leadership in prevention and public education; conducts research and teaching.**Level II**Able to initiate definitive care for all injured patients; 24 h immediate coverage by general surgeons and coverage by specialties such as orthopedic surgery, neurosurgery, anesthesiology, emergency medicine, radiology, and critical care; provides trauma prevention and continuing education programs.**Level III**Provides prompt assessment, resuscitation, surgery, intensive care, and stabilization of injured patients; 24 h immediate coverage by emergency medicine physicians and prompt availability of general surgeons and anesthesiologists; has transfer agreements for patients requiring more comprehensive care.**Level IV**Provides advanced trauma life support (ATLS) prior to transfer; basic emergency department facilities to implement ATLS protocols and 24 h laboratory coverage; may provide surgery and critical care services if available.**Level V**Provides initial evaluation, stabilization, and diagnostic capabilities; prepares patients for transfer to higher levels of care; basic emergency department facilities to implement ATLS protocols.

## 5. Results

Table 1 summarizes trends in hospital ED and trauma center capacity along with population growth and aging between 2003–2023.

**Population trends:** California’s overall population increased by 11% between 2003 and 2023, from 35.25 million to 39.2 million. The population aged 65 and older increased significantly, growing by 70% from 3.7 million to 6.3 million. This demographic shift increased the percentage of the population aged 65 and older from 10% in 2003 to 16% in 2023.

**Hospitals and emergency departments:** The total number of general acute care hospitals in California decreased slightly by 5%, from 398 in 2003 to 379 in 2023. The number of hospitals with emergency departments also decreased by 5%, from 338 to 320. Hospitals with emergency departments but without trauma centers showed the most significant decline, decreasing by 16%, from 283 to 238. In contrast, hospitals with both emergency departments and trauma centers increased substantially by 51%, from 55 in 2003 to 83 in 2023.

**Licensed beds:** The total number of licensed beds in general acute care hospitals decreased slightly by 2%, from 80,621 in 2003 to 78,902 in 2023. The number of licensed beds in hospitals with emergency departments showed a marginal increase of 1%, from 73,163 to 73,606. However, hospitals with emergency departments but without trauma centers experienced a 12% decrease in licensed beds, from 54,015 to 47,605. Conversely, hospitals with emergency departments and trauma centers saw a significant increase in licensed beds, growing by 36% from 19,148 to 26,001.

**ED stations:** The total number of ED stations in hospitals with emergency departments increased substantially by 56%, from 5592 in 2003 to 8711 in 2023. Hospitals with emergency departments but without trauma centers showed an increase in ED stations, rising by 36% from 4198 to 5690. The largest growth occurred in hospitals with emergency departments and designated trauma centers, where the number of ED stations more than doubled, increasing by 115% from 1394 to 3003.

**Percentage of hospitals with an ED or ED + trauma center:** The percentage of general acute care hospitals with emergency departments remained relatively stable, decreasing slightly from 85% in 2003 to 84% in 2023. However, the percentage of all general acute care hospitals with trauma centers increased from 14% in 2003 to 22% in 2023. The percentage of hospitals with emergency departments that also have a trauma center rose from 16% to 26%. Additionally, the percentage of all beds located in hospitals with trauma centers increased from 24% to 33%, and the percentage of all ED stations in hospitals with trauma centers increased from 25% to 34%. It is important to note that, in 2003, only 22 of California’s 58 counties had an in-county designated trauma center; by 2023, that number had risen to 30 counties. This eight-county increase reflects expanded trauma services into areas that previously relied on out-of-county transfers.

Table 2 summarizes trends in hospital inpatient utilization, including inpatient admissions, ED visits, and inpatient admissions via hospital EDs.

**Total inpatient admissions:** Despite the population increasing and aging, total inpatient admissions in California decreased. For all general acute care hospitals, admissions decreased by 8%, from 3,365,315 in 2003 to 3,107,461 in 2023. Hospitals with emergency departments also experienced a decrease in total inpatient admissions, with a 7% reduction from 3,207,137 to 2,988,462. The largest reduction in inpatient admissions occurred in hospitals with emergency departments but without trauma centers, which saw a 22% decrease from 2,375,674 to 1,854,980. In contrast, hospitals with both emergency departments and trauma centers experienced a substantial increase in inpatient admissions, rising by 36% from 831,463 to 1,133,482.

**ED visits:** In contrast to the decline in overall inpatient admissions, ED visits increased significantly. For hospitals with emergency departments, ED visits increased by 49%, from 9,864,180 in 2003 to 14,691,817 in 2023. Hospitals with emergency departments but without trauma centers saw a 29% increase in ED visits, from 7,551,595 to 9,744,947. However, hospitals with emergency departments and trauma centers experienced the most dramatic growth in ED visits, with a 114% increase from 2,312,585 to 4,946,870.

**ED visits resulting in inpatient admission:** The number of ED visits resulting in inpatient admission also increased substantially. Among hospitals with emergency departments, these admissions increased by 48%, from 1,357,324 in 2003 to 2,012,973 in 2023. Hospitals with emergency departments but without trauma centers saw a 29% increase in ED visits resulting in inpatient admissions from 1,011,782 to 1,303,292. Hospitals with emergency departments and trauma centers showed the largest increase, with a 105% rise from 345,542 to 709,681.

Table 3 summarizes the trends in capacity and utilization between 2003 and 2023 for hospitals with designated trauma centers. There are four (4) levels of designation as defined by the Emergency Medical Services Authority (EMSA). Level I trauma centers offer the highest level of trauma care, providing comprehensive treatment from prevention through rehabilitation. They have 24/7 specialized personnel and resources, conduct research, and offer education. Level II trauma centers are similar to Level I, but are not mandated to engage in research or offer extensive education. They provide comprehensive trauma care with the immediate availability of essential specialties, personnel, and equipment. Level III trauma centers can assess and stabilize patients with traumatic injuries and provide prompt surgical intervention. They typically have transfer agreements for patients needing more comprehensive care. Level IV trauma centers provide advanced trauma life support before transfer. They offer initial evaluation, stabilization, and diagnostics, with established transfer agreements for extensive care needs.

**Growth in emergency department capacity:** Between 2003 and 2023, the number of hospitals with trauma center EDs increased from 59 to 83 (41% rise). This was driven by growth in Level II, III, and IV hospitals, while Level I hospitals declined. Hospitals with pediatric ED designations also increased, especially Level I and II-Pediatric. The number of ED stations more than doubled (115% increase) from 1394 to 3003. Lower-tier trauma centers expanded the most, with Level III and IV hospitals increasing by 164% and 723%, respectively. Pediatric-designated ED stations also grew, with Level I and II-Pediatric hospitals adding 301 stations.

Table 3 summarizes capacity and utilization, comparing 2003 and 2023, for hospitals with trauma centers, by level (I-IV) and type (Adult, Pediatric) of trauma center designation.

**Utilization of emergency departments:** Total ED visits increased sharply by 114%, from approximately 2.3 million in 2003 to nearly 4.95 million in 2023, particularly in lower-tier trauma centers. Level IV hospitals had a 914% increase in visits, indicating greater reliance on lower-level emergency care. Level III hospitals experienced a 133% rise. In contrast, Level I trauma center visits declined by 20%, suggesting a shift toward lower-tier hospitals. Pediatric-designated hospitals showed the most dramatic growth, with ED visits surging by 553% to nearly 1.38 million in 2023, highlighting increased demand for specialized pediatric emergency care.

In summary, over the 20+ year study period, ED capacity and ED-based utilization have substantially increased. The number of hospitals with trauma centers rose by 41%, and ED stations within them more than doubled. ED visits in trauma hospitals surged by 114%, with the largest increases in lower-tier and pediatric-designated hospitals. While Level I trauma center visits decreased, Level IV facilities saw a 914% surge, indicating a shift in emergency care reliance. Pediatric emergency services expanded significantly.

**Figure 1:**
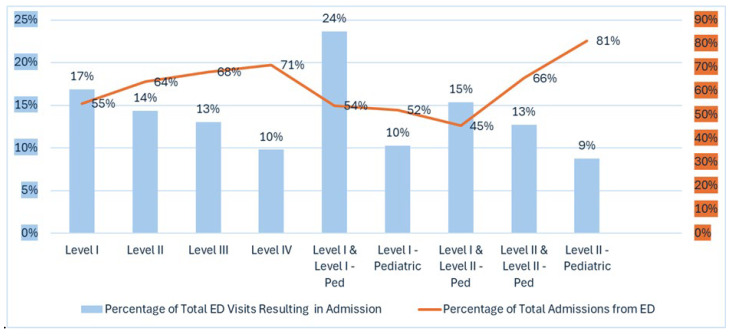
Percentage of total ED visits resulting in inpatient admission and percentage of total inpatient admissions via EDs within trauma center hospitals by level of trauma designation. Blue coded values on left axis indicate % Total ED Visits Resulting in Admission and Orange coded values on right axis Indicate % of Total Admissions from ED.

Figure 1 illustrates admission patterns across hospitals by trauma designation level, revealing clear utilization trends.

**ED to admission rate:** Level I and Level I-Pediatric centers have the highest ED to admission rate (24%), while Level I centers alone admit 17% of ED visits. Lower-level trauma centers have lower rates, with Level IV at 10% and pediatric specialty centers at 9%. Higher-level trauma centers (especially Level I and Level I-Pediatric) have higher rates (24%), indicating they treat more complex cases requiring hospitalization, while lower-level and pediatric-specific centers have lower rates (9–10%).

**Percentage of total admissions via EDs:** The percentage of admissions originating from EDs varies. Level II-Pediatric centers have the highest percentage (81%), while Level I and Level II-Pediatric centers have the lowest (45%), suggesting more transfers or scheduled admissions from other sources.

**Figure 2:**
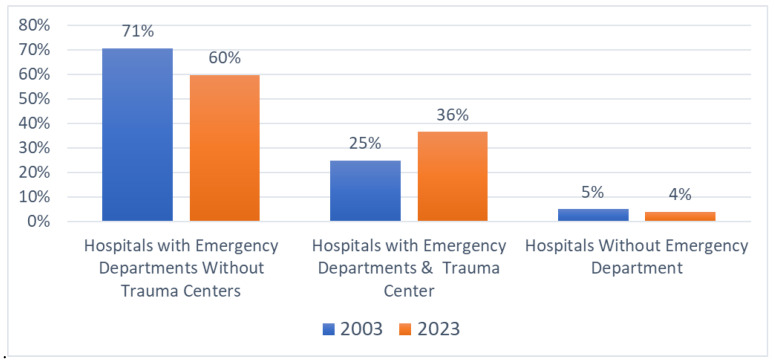
Percentage of total inpatient admissions via EDs by hospital type, 2003 and 2023.

Figure 2 summarizes the percentage of total inpatient admissions across the state originating in hospitals based on ED and trauma service availability.

Between 2003 and 2023, hospitals with both EDs and trauma centers (TCs) significantly increased their share of total statewide inpatient admissions from 25% to 36% (an 11-percentage-point increase), highlighting the growing importance of trauma centers. Concurrently, hospitals with EDs but without trauma center designation decreased their share from 71% to 60%, while hospitals without EDs maintained a stable but small portion (from 5% to 4%).

## 6. Discussion

In this section, we summarize our key findings and draw upon and integrate the relevant scientific literature to discuss the applications, implications, and contributions of our findings to the literature and to healthcare policy more broadly. We focus our discussion on several key areas, including the growing challenge of healthcare affordability in the USA, the structure and impact on Value-Based Care (VBC) models designed to improve healthcare outcomes and affordability, and, finally, the potential inflationary effects of the expansion of hospital-based trauma centers within the hospital sector on hospital prices and healthcare affordability in the USA. Our findings highlight several key trends:

(1) Hospital-based emergency department (ED) and trauma center capacities have increased significantly, making these services a larger part of the healthcare delivery system;
(2) A majority and increasing share of inpatient admissions in hospitals with EDs originate through the ED;
(3) A significant and growing portion of system-wide ED visits and inpatient admissions occur in hospitals with trauma centers.

These trends have major implications for health system efficiency, healthcare spending growth, and affordability in the USA.

### 6.1. The Challenge of Healthcare Affordability in the US

The United States healthcare system faces escalating costs, with resulting challenges to affordability and broader economic impacts [11]. In 2021, national health expenditures reached USD 4.3 trillion, representing 18.3% of GDP, a rate higher than most other developed nations [1]. This spending averages USD 12,914 per person, and yet many Americans still struggle to access needed care and manage healthcare bills. A key factor is the misalignment of incentives in the fee-for-service model, where providers are paid based on service volume rather than patient outcomes, contributing to these persistent issues [12,13].

To address these problems, Value-Based Care (VBC) models have emerged as alternatives [14,15,16,17,18]. VBC links payment to the quality and results of care, aiming to improve health outcomes relative to total costs [19,20]. This discussion explores the goals and challenges of VBC adoption, especially regarding how increased emergency department and hospital-based trauma center use may hinder VBC’s effectiveness in improving outcomes and affordability [21]. Understanding these dynamics is essential for stakeholders seeking a more sustainable, patient-centered healthcare system [22].

### 6.2. Adoption of Value-Based Care Models to Improve Outcomes and Affordability: Goals and Challenges

Employers, payors, and policymakers are pursuing reforms to improve health system outcomes and control spending [14]. Value-Based Care (VBC) models are expanding in the USA, aiming to enhance value and affordability by improving care quality and moderating cost growth. These models shift the focus from service volume to high-quality, efficient, and equitable care for patients and providers. VBC emphasizes managing patient care across services, prioritizing prevention, and reducing avoidable emergency admissions. Instead of fee-for-service, VBC links provider incentives to quality, outcomes, and cost-effectiveness [12]. The objectives include better patient experience, health equity, improved population health, optimized spending, and workforce well-being [14]. Central to VBC is longitudinal patient-centered care that seeks to preempt costly acute episodes through proactive, coordinated interventions [13,14,15,16,17].

### 6.3. Rising Emergency Department Admissions Challenge Value-Based Care Effectiveness

The increasing share of hospital admissions from EDs, as shown in our findings and national data, signals a potential breakdown in VBC’s preventive mechanisms and challenges its effectiveness in improving affordability [18,19]. Greater reliance on costly ED-based acute care can undermine VBC’s cost-saving goals and burden providers with downside risk contracts tied to quality and cost benchmarks. Persistent high ED-based admissions highlight the need for VBC frameworks to address factors driving acute episodes requiring emergency care [20,21,22]. Because emergency care is much more expensive than comparable primary or urgent care, higher ED admissions impact the cost savings VBC aims to achieve [23]. This can negatively affect provider performance and financial incentives under VBC contracts. Addressing this requires managing the drivers of increased ED admissions, possibly by integrating EDs into VBC networks, enhancing prevention, and improving outpatient access. Increased ED reliance, focused on acute interventions, disrupts VBC principles by creating care discontinuities and bypassing mechanisms for managing health across the continuum [20,21]. Structural misalignments from increased ED admissions create obstacles to coordination, chronic disease management, and cost containment [17]. Thus, VBC’s effectiveness depends on adapting to increased ED use, potentially by formally integrating EDs into the value chain and investing in data integration, analytics, and transitional care [24].

### 6.4. Fragmentation and Discontinuity in Care Due to Emergency Department Admissions

EDs often function outside integrated care management, creating care fragmentation and discontinuity [25]. This can result in redundant testing, care gaps, and poor communication during transitions, undermining VBC principles of continuity and coordination. EDs, once mainly for triage and stabilization, now handle complex diagnostics and inpatient admissions, roles previously managed by primary care [26,27]. This shift challenges VBC, as EDs may lack seamless access to patient records or established care plans [25]. Fragmentation can lead to duplicative testing, missed follow-ups, and poor transition communication, impeding VBC goals and causing inefficiencies and suboptimal outcomes [17]. Emergency admissions often occur without input from the patient’s primary care team, reducing the benefits of relationship-based care and undermining VBC’s informational foundation [28].

### 6.5. Integrating Expanded Role of EDs and Trauma Centers into VBC Models

To align rising ED use and the expanding role of trauma centers with VBC, systemic and structural changes are needed [29]. Integrating EDs into VBC networks using shared EHRs, real-time data, and unified protocols is one solution [29,30,31]. This would give ED clinicians access to histories, improve adherence to care plans, and strengthen post-discharge continuity. Predictive analytics can identify high-risk patients for proactive interventions, such as enhanced primary care or community support. Effective discharge planning and timely outpatient follow-up are vital to prevent avoidable readmissions and reduce ED dependence as a hospital entry point. The rise in ED-driven hospitalizations both challenges VBC and prompts its evolution, revealing gaps in outpatient care, data interoperability, and patient engagement. Future success depends on aligning quality metrics across settings and investing in interoperable health data. Enhancing health literacy and encouraging appropriate care-seeking are also essential. As payment models reward value, EDs must become strategic hubs in coordinated care, not isolated cost centers. Incorporating emergency care into VBC is crucial for achieving efficiency, equity, and improved population health [19,32].

Another important finding from this research is that a significant and increasing share of system-wide ED visits and inpatient admissions now occur in hospitals with trauma centers. The core goals of Value-Based Care (VBC)—improving patient outcomes while controlling costs—face substantial challenges from current market structures, particularly the role and pricing power of designated trauma centers. While VBC initiatives seek to incentivize efficiency and prevention to reduce costly acute episodes, the growing concentration of hospital services within trauma centers introduces complex market dynamics that can drive prices higher, potentially undermining VBC objectives, including affordability. A recent 2024 Health Affairs study provides critical insight into this issue [33]. The researchers examined whether the market power granted to trauma centers affects the pricing of services unrelated to trauma. Trauma centers often have effective monopolies over trauma services in specific areas due to regulatory restrictions on new entry and protocols requiring trauma patients to be transported to designated facilities. These rules are intended to ensure sufficient patient volume for quality and efficiency. However, Kessler et al. hypothesized that this monopoly power could be leveraged to negotiate higher prices for non-trauma care. Analyzing commercial claims data for about 2000 hospitals from 2012 to 2018, the study compared prices at trauma centers versus non-designated hospitals for non-trauma inpatient admissions and ED visits. After controlling for confounders and hospital fixed effects, the study found that trauma centers charged significantly higher prices for non-trauma inpatient admissions (about 4% higher) and non-trauma ED visits (about 3–5% higher) than hospitals without the trauma designation.

These findings of trauma center pricing power are especially important when combined with our results, which show growth in trauma centers and a rising share of non-trauma inpatient use at these hospitals.

This expanding role means the identified price premium at trauma centers applies to a larger segment of overall hospital care. The implications for healthcare costs and affordability are significant. Trauma centers’ ability to leverage market power in trauma services to extract higher prices in other areas contributes directly to rising hospital prices and insurance premiums in the commercial market. This dynamic poses a challenge for policymakers trying to balance access to high-quality trauma care with controlling overall healthcare spending and improving affordability. Health economists argue that, to address market failure, new payment policies should directly target high prices, either as a trigger for regulation or as a screening tool [34]. The success of VBC models in containing costs may be limited unless strategies are developed to address or account for the pricing power of influential, essential hospital providers like trauma centers [35].

### 6.6. Limitations

While this study relies on self-reported data by each hospital included in the study, the data reporting systems that the hospitals used to submit their data have been operational for more than 35 years. This has allowed for the system to be fully implemented and widely used by hospitals, government agencies, and researchers for many years, providing increased confidence in the data. The data are also reviewed by the government agency, HCAI, for completeness and consistency before being accepted for publication on the agency’s website. In addition, since our analytical approach is primarily descriptive analysis of data aggregated across all or large sub-sets of hospitals, hospital variance due to hospital-level reporting errors is likely to be diminished. In addition, while our data are from a single state in the USA—California—the sample is large and represents a wide cross-section of hospitals with different characteristics, and thus is likely to be reasonably representative of general acute care hospitals in the USA.

## 7. Conclusions

In conclusion, our findings on the expanding role of emergency departments (EDs) and trauma centers in the healthcare delivery system have profound implications for initiatives such as Value-Based Care (VBC) designed to improve outcomes and affordability. The documented trends—significant growth in hospital-based EDs and trauma center capacity, increasing proportion of inpatient admissions originating through EDs, and growing concentration of system-wide ED visits and admissions in trauma centers—represent a fundamental shift in how patients access hospital care and in the role of hospitals within the overall healthcare system. This combination of forces creates both a challenging environment for VBC models and other policy initiatives that seek to align provider incentives with quality outcomes and cost efficiency, as well as improve affordability, and necessitates further research to support these efforts. And, finally, while not the main focus of this paper, given the growth in both EDs and trauma utilization, it will be important to expand policy discussions and research relative to long-term planning to ensure an adequate supply of emergency medicine manpower, including physicians and other emergency-trained personnel.

## Figures and Tables

**Table 1:** Demographics and hospital ED and trauma center capacity, 2003 and 2023.

Population	2003	2023	% Change
Population—total (millions)	35.25	39.2	11%
Population, age 65+ total (millions)	3.7	6.3	70%
Population, age 65+, percentage total of population	10%	16%	-
**Hospitals—Number**	**2003**	**2023**	**% Change**
All hospitals general acute care (GAC)	**398**	379	−5%
Hospitals with emergency departments	**338**	320	−5%
Hospitals with emergency departments without trauma centers	**283**	238	−16%
Hospitals with emergency departments and trauma centers	**55**	83	51%
**Licensed Beds—Number**	**2003**	**2023**	**% Change**
All hospitals general acute care	80,621	78,902	−2%
Hospitals with emergency departments	73,163	73,606	1%
Hospitals with emergency departments without trauma centers	54,015	47,605	−12%
Hospitals with emergency departments and trauma centers	19,148	26,001	36%
**ED Stations—Number**	**2003**	**2023**	**% Change**
All hospitals with emergency departments	5592	8711	56%
All hospitals with emergency departments without trauma centers	4198	5690	36%
All hospitals with emergency departments and designated trauma centers	1394	3003	115%
**Percentage Hospitals with ED or ED + Trauma Center**	**2003**	**2023**	
% Total GAC hospitals with EDs	85%	84%	
% of All GAC hospitals with trauma centers	14%	22%	
% GAC hospitals with EDs that also have a trauma center	16%	26%	
% All beds in hospitals with trauma centers	24%	33%	
% All ED stations in hospitals with trauma centers	25%	34%	

**Table 2:** Hospital inpatient and ED utilization, 2003 and 2023.

	2003	2023	% Change
**Total Inpatient Admissions**			
All hospitals general acute care	3,365,315	3,107,461	−8%
Hospitals with EDs	3,207,137	2,988,462	−7%
Hospitals with EDs without trauma centers	2,375,674	1,854,980	−22%
Hospitals with EDs and trauma centers	831,463	1,133,482	36%
**ED Visits**			
Hospitals with EDs	9,864,180	14,691,817	49%
Hospitals with EDs without trauma centers	7,551,595	9,744,947	29%
Hospitals with EDs and trauma centers	2,312,585	4,946,870	114%
**ED Visits Resulting in IP Admission**			
Hospitals with EDs	1,357,324	2,012,973	48%
Hospitals with EDs without trauma centers	1,011,782	1,303,292	29%
Hospitals with EDs and trauma centers	345,542	709,681	105%

**Table 3:** Capacity and utilization in hospitals with trauma centers by level of trauma designation, 2003 and 2023.

	Number of Hospitals	Number of Hospitals		ED Stations	ED Stations		ED Visits Total	ED Visits Total	
**Level Trauma Designation**	**2003**	**2023**	**% Change**	**2003**	**2023**	**% Change**	**2003**	**2023**	**% Change**
**Level I**	11	8	−27%	386	419	9%	692,097	551,974	−20%
**Level II**	28	31	11%	721	1199	66%	1,142,315	2,146,965	88%
**Level III**	10	14	40%	139	367	164%	235,117	547,509	133%
**Level IV**	5	12	140%	22	181	723%	31,309	317,510	914%
**Level I and Pediatric I**	1	4	300%	42	159	279%	51,598	207,540	302%
**Level I and Level II-Pediatric**	0	5	-	-	301	-	-	377,816	-
**Level II and Pediatric-II**	0	3	-	-	145	-	-	282,494	-
**Level I-Pediatric**	1	4	300%	25	157	528%	56,988	313,599	450%
**Level II-Pediatric**	3	2	−33%	59	75	27%	103,161	201,463	95%
**TOTAL**	59	83	41%	1394	3003	115%	2,312,585	4,946,870	114%
**Summary**									
**Level 1–4—No Pediatric**	54	65		1268	2166		2,100,838	3,563,958	70%
**Level 1–4 + Pediatric**	5	18		126	837		211,747	1,382,912	553%
**TOTAL**	59	83		1394	3003		2,312,585	4,946,870	114%

## Data Availability

This study uses publicly available data from the Department of Health Care Access and Information (HCAI), California Health and Human Services. Hospital annual utilization report and pivot tables. https://data.chhs.ca.gov/dataset/hospital-annual-utilization-report, (accessed on January 11 2024). The data are anonymized and open access, and the data were managed to ensure the safety of any protected fields. Data are publicly available at the above link for access by the public and researchers. The agency collecting the data has a process that ensures the safety of protected fields.
